# Prevalence and characteristics of the aberrant anterior tibial artery: a single-center magnetic resonance imaging study and scoping review

**DOI:** 10.1186/s12891-021-04801-9

**Published:** 2021-11-02

**Authors:** Julio Marin-Concha, Pablo Rengifo, Pedro Tapia, Daniel Kaiser, Timo Siepmann

**Affiliations:** 1 Radiology Department Clínica SANNA, San Borja, Lima Peru; 2 Division of Health Care Sciences Center for Clinical Research and Management Education Dresden, Dresden International University, Dresden, Germany; 3 Institute of Diagnostic and Interventional Neuroradiology, University Hospital Carl Gustav Carus, Technische Universität Dresden, Dresden, Germany; 4 Department of Neurology, University Hospital Carl Gustav Carus, Technische Universität Dresden, Dresden, Germany

**Keywords:** Popliteal artery, Aberrant anterior tibial artery, MRI, Anatomical variations, Knee

## Abstract

**Background:**

Planning surgical procedures of the lower leg benefits from considering the possibility of an aberrant anterior tibial artery (AATA), but previously published data on the frequency of this anatomic variant shows heterogeneity. We assessed the prevalence of AATA in a Latin American cohort using magnetic resonance imaging (MRI) and compared these with other studies reported in the literature.

**Methods:**

We retrospectively included consecutive patients who had undergone multiplanar knee MRI at a radiology department in Lima, Peru. The MRI protocol included coronal T1 weighted, axial, sagittal and coronal proton density fat-saturated (PDFS) and sagittal T2 weighted images.

Two experienced radiologists assessed all images and were blinded to each other’s findings. The frequency of the AATA was compared to previous cohorts. A scoping review was undertaken to provide an overview of previously published data on the prevalence of ATAA.

**Results:**

We analyzed 280 knee MRI examinations of 253 patients (median age 41 years (IQR 31–52), 53.8% male). The aberrant anterior tibial artery variant was present in 8 of 280 (2.9%) evaluated knees, resulting in a prevalence of 3.2% in our study population. The PDFS sequence in the axial or sagittal orientation was most effective to identify AATA. The frequency of AATA in the reviewed literature using different radiological modalities ranged from 0.4 to 6% (median 1%, IQR (0.5–2.3%).

**Conclusions:**

The AATA is a frequent vascular variant that can be detected by MRI in the preparation of invasive interventions of the lower leg.

**Supplementary Information:**

The online version contains supplementary material available at 10.1186/s12891-021-04801-9.

## Background

**Fig. 1:**
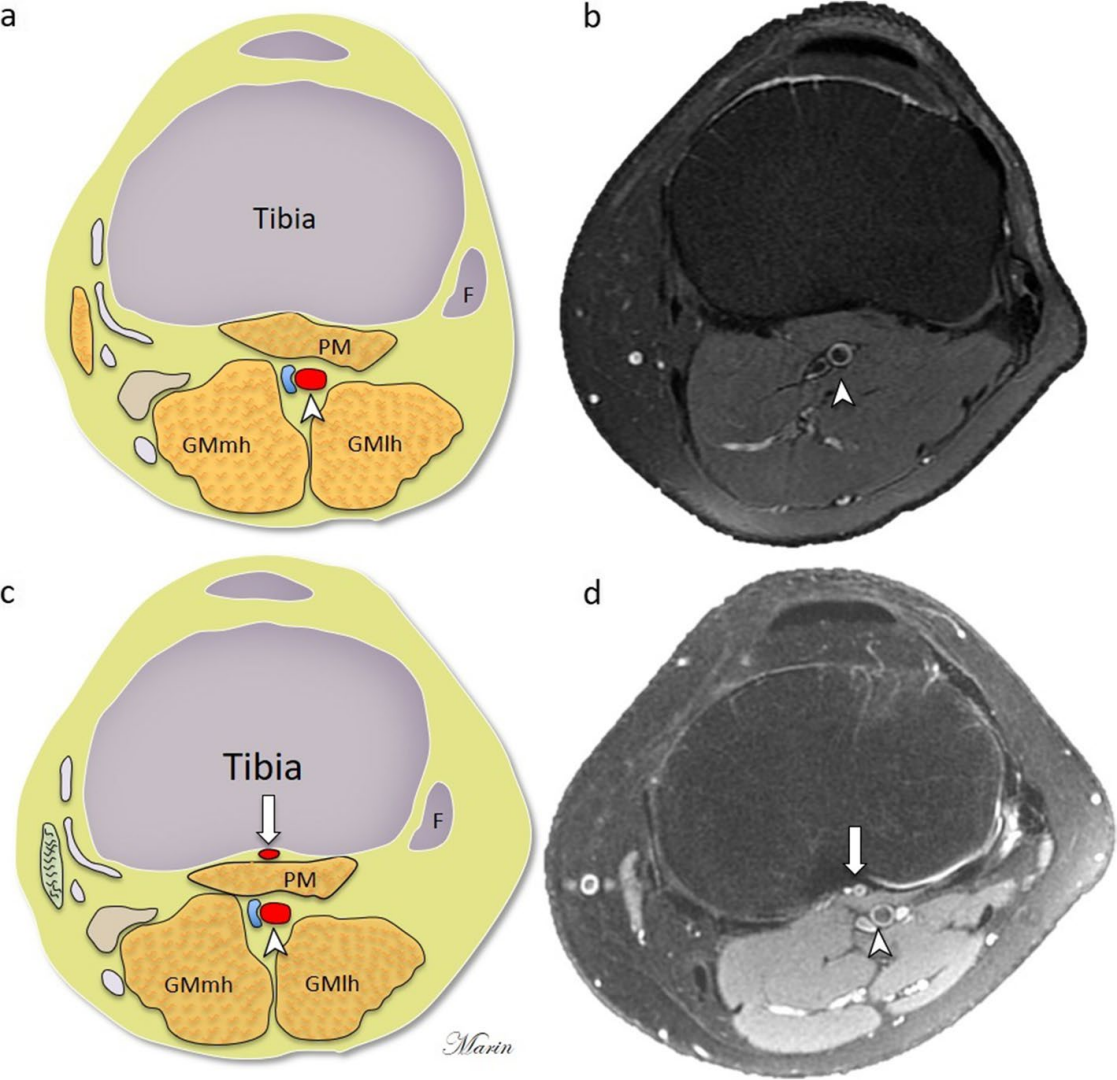
**a**, **b** Schematic axial diagram of normal knee anatomy and Axial PDFS MRI of normal knee anatomy showing the popliteal artery (arrowhead) located posterior to the popliteal muscle (PM). **c**, **d** Schematic axial diagram and Axial PDFS MRI of the knee that show the aberrant anterior tibial artery (arrow) between the tibial cortex and the popliteal muscle (PM). Fibula (F), Gastrocnemius muscle media head (GMmh), Gastrocnemius muscle lateral head (GMlh). Figure created by J. Marin-Concha

**Fig. 2:**
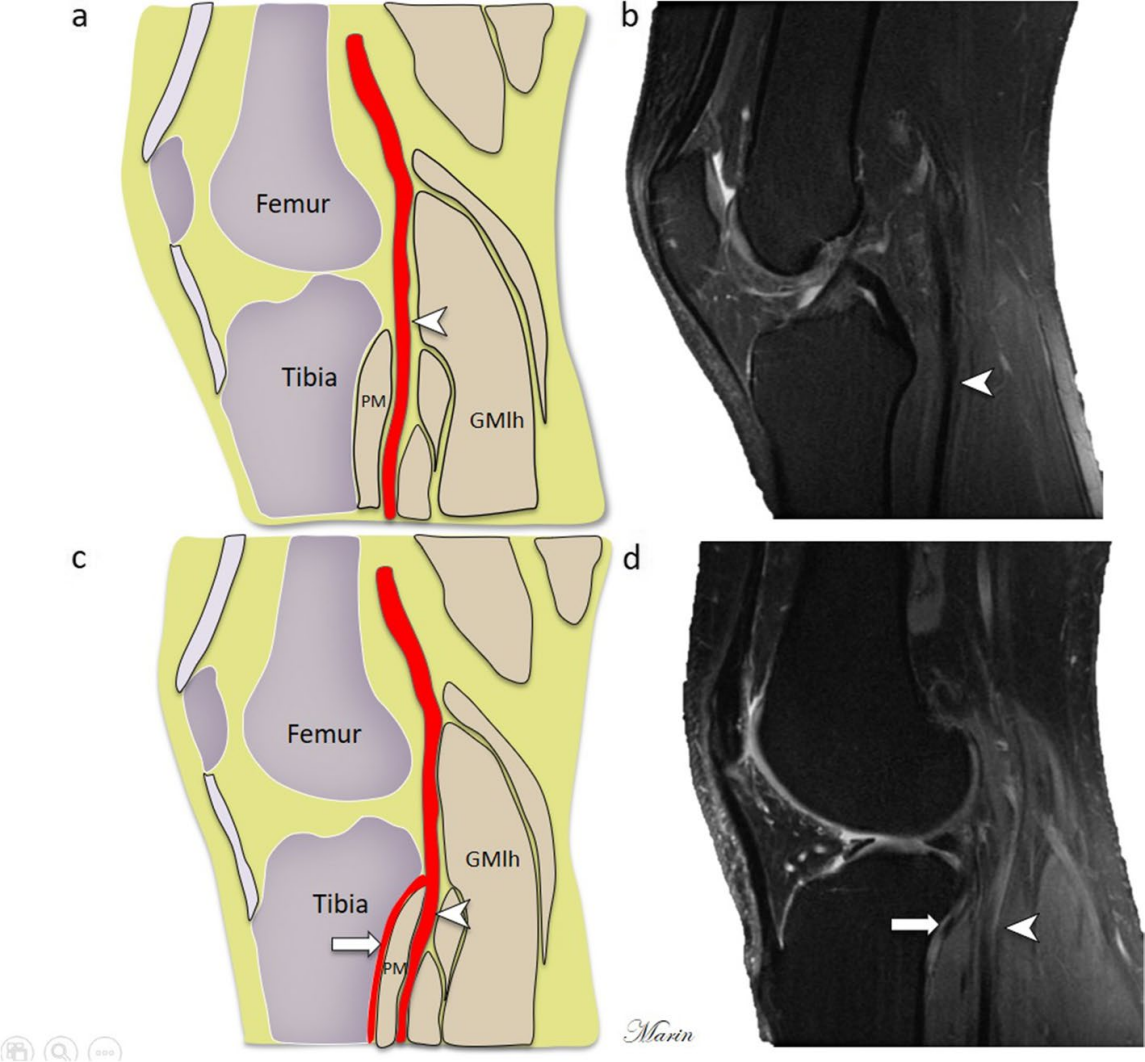
**a**, **b** Schematic sagittal diagram of normal knee anatomy and sagittal PDFS MRI of normal knee anatomy show the popliteal artery (arrowhead) located posterior to the popliteal muscle (PM). **c**, **d** Schematic sagittal diagram and sagittal PDFS MRI of the knee that show the aberrant anterior tibial artery (arrow) between the tibial cortex and the popliteal muscle (PM). Fibula (F), Gastrocnemius muscle media head (GMmh), Gastrocnemius muscle lateral head (GMlh). Figure created by J. Marin-Concha

The anatomic variations of popliteal artery terminal branching are relatively common with a prevelance of around 10% of limbs and should be considered by radiologists and surgeons [1]. Vascular injuries during invasive procedures are rare but can cause serious complications such as arterial transection, pseudoaneuryms and limb loss [2–4]. The popliteal artery is the continuation of the femoral artery after it passes through the adductor hiatus, and it normally ends at the inferior border of the popliteus muscle where it splits into the anterior tibial artery and the tibioperoneal trunk [5]. Previous research has reported different types of anatomical variations of popliteal terminal branching such as the high origin of the anterior tibial artery, which manifests when the vessels split above the knee joint or at the upper part of the popliteus muscle [6]. The aberrant anterior tibial artery (AATA) is a subtype variant in which the artery courses between the popliteal muscle and the tibial cortex (Figs. 1 and 2). This variant has been reported in autopsy, angiography, ultrasound and computed tomography (CT) angiography studies [6–8]. However, data on frequency vary, and the capacity of magnetic resonance imaging (MRI) to detect this variant is not widely reported [9]. Using MRI to assess AATA may provide an adequate report for the referral physician especially if surgery is planned. We aimed to assess the frequency and magnetic resonance characteristics of the AATA in a Latin American cohort and compared our findings with previous research done with other imaging modalities by a scoping review.

## Methods

### Subjects, protocol

We evaluated MRI examinations of the knee of consecutive patients who had been assessed at our radiology department in Lima, Peru (Radiology Department Clínica SANNA, San Borja, Lima, Peru) from October 2018 to December 2018. To enhance external validity we did not restrict any indications of MRI in the selection of subjects. Our exclusion criteria were an inadequate knee MRI evaluation due to motion artifact or theinability of the patient to complete the test. The need for ethics approval and consent was waived by the Institutional Review Boards of Clinica San Borja and the Universidad Peruana Cayetano Heredia (19-6681) due to the retrospective nature of the study. We obtained permission from Clinica SANNA, San Borja to get access to the clinical and imaging patient data. Patient characteristics were recorded according to the clinical history.

### Image acquisition and analysis

Images were acquired on a 1.5 Tesla Optima MR450 and 1.5 Tesla Optima MR430s (GE Healthcare, Waukesha, Wisconsin, USA) using a dedicated 16 channel knee coil. The MRI protocol included acquisitions of proton density fat-saturated (PDFS) weighted images in coronal (TR/TE = 3100/35; echo train length, 8; 3 mm thickness; space between slices 0.5 mm), sagittal (TR/TE = 3300/35; echo train length, 8; 2 mm thickness; space between slices 0.5 mm) and axial (TR/TE = 3300/35; echo train length, 8; 3.2 mm thickness; space between slices 0.5 mm), coronal T1 weighted image (TR/TE = 600/12; echo train length, 2; 3 mm thickness; space between slices 0.5 mm) and sagittal T2 weighted images (TR/TE = 4360/90; Echo train length, 20; 2 mm thickness; space between slices 0.5 mm). Two experienced radiologists reviewed the knee MRI examinations and determined the presence or absence of this anatomical variation. Both radiologists were blinded to each other’s findings. Cases of AATA were reviewed again by both radiologists for consensus.

The anatomical variant of AATA was classified as shown in Figs. 1 and 2.

### Scoping review

We conducted a scoping review using Medline, PubMed and PubMed Central (PMC), Google Scholar, Scopus and Embase databases to identify studies examining the frequency of the AATA in the literature. Eligibile studies included those conducted via anatomical dissection, ultrasound, CT, angiography and MRI of unilateral and bilateral knees of cadeveric and individuals without knee pathologies. The literature search was performed by two reviewers (JM, PR) between February 2021-June 2021 including articles published from 1928 to 2020 in English. The following MESH terms were included in the approach using Boolean operators “AND” and “OR” in Pubmed: “Popliteal artery”, “Knee”, “magnetic resonance imaging”, “angiography”, “computer tomography”, “Doppler ultrasound”. Further details are shown in Supplement 1. Six hundred and seventy five studies were detected using the term Mesh and search limits. One hundred and twenty five abstracts were screened for elegibility, and duplicates were excluded manually. Reference lists of the selected articles were manually reviewed for additional articles. Ultimately, 20 studies provided information identifiying the frequency of AATA. Data were reported per the guidelines established by the Preferred Reporting Items for Systematic reviews and Meta-analyses extension for scoping reviews (PRISMA-ScR) [10]. The data extraction was performed by two reviewers (JM, PR). The data that was extracted included the following: year of publication, location of study, study design, type of study, level of evidence, patient demographics, journal type and funding. Data was synthesized for frequency of the AATA. Study quality was assessed using The Newcastle-Ottawa Scale (NOS) [11] for assessing the quality of nonrandomized studies in order to provide a measure of robustness of the current body of evidence on the AATA with a focus on: selection of the study groups (representativeness of exposed cohor, selection of non-exposed cohort, ascertainment of exposure, demonstration that outcome of interest was not present at start of study); comparability of the groups (adjust for the most important factors and other risk factors); and the ascertainment of either the exposure or outcome (Assessment of outcome, follow-up length and loss to follow-up rate). Good quality studies obtain 3–4 stars in the selection domain, 1–2 in the comparability domain and 2–3 in the exposure domain, Fair quality studies obtain 2 stairs in the selection domain, 1–2 in comparatibility and 2–3 outcome domain. Finally poor studies obtain 0–1 in all the domains.

### Statistical analysis

We registered all data and performed descriptive statistics using Microsoft Office Excel 2016, Los Angeles, California, USA. The kappa test of agreement was used to determine agreement for the recognition of the AATA. A kappa value of 0.2 was defined as slight agreement; 0.21 – 0.40 as fair agreement; 0.41 – 0.60 as moderate agreement; 0.61–0.80 as strong agreement, and 0.81 – 1.00 as almost perfect agreement.

## Results

### Patients

We reviewed 280 consecutive knee MRI examinations of 253 patients. The median age of the patients was 41 years (interquartile range 31–52), and 136 (53.8%) were males. In total, we examined 147 (52.6%) right and 133 (47.5%) left knees with an evaluation of both knees in 27 patients.

### Magnetic resonance imaging study

**Fig. 3:**
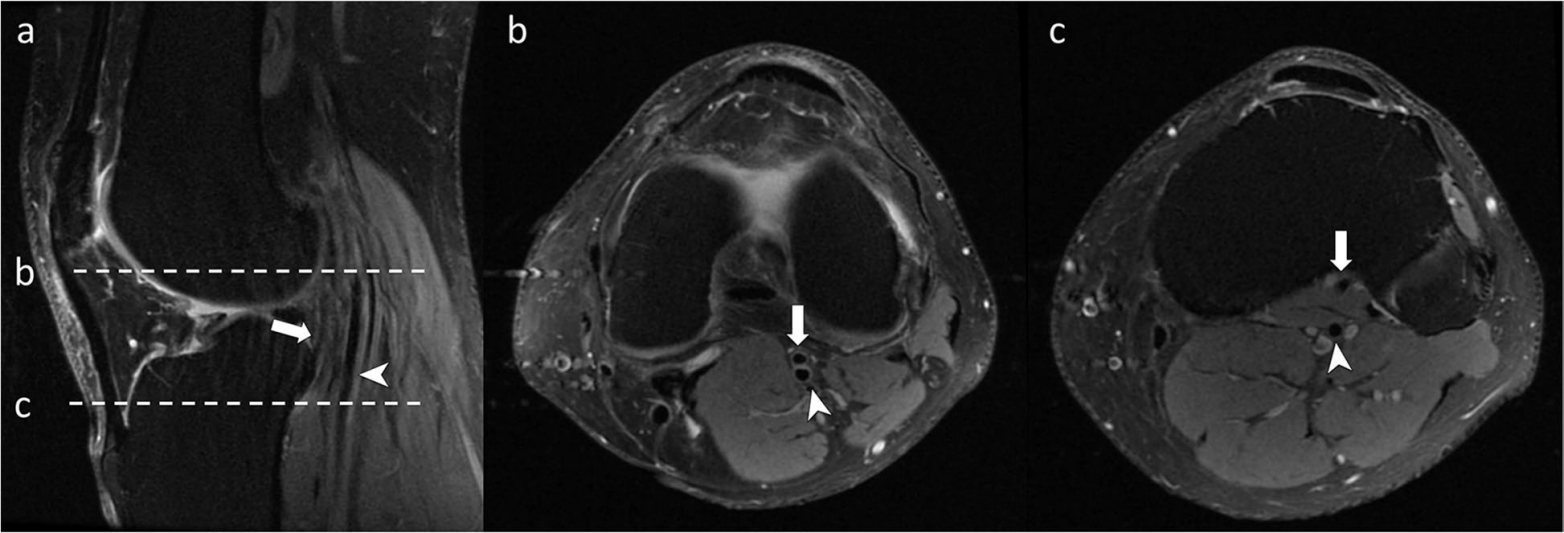
A male patient (age range 45–65) with an aberrant anterior tibial artery. **a** Sagittal PDFS fat saturation shows the popliteal artery (arrowhead) running anteriorly to the popliteus muscle and the aberrant anterior tibial artery (arrow) posterior to the tibial cortex. **b** Axial PDFS image just below the origin of the aberrant anterior tibial artery. **c** Distribution of the aberrant anterior tibial artery and the popliteal artery at the upper part of the tibia. This is the typical location of a high tibial osteotomy surgery

**Fig. 4:**
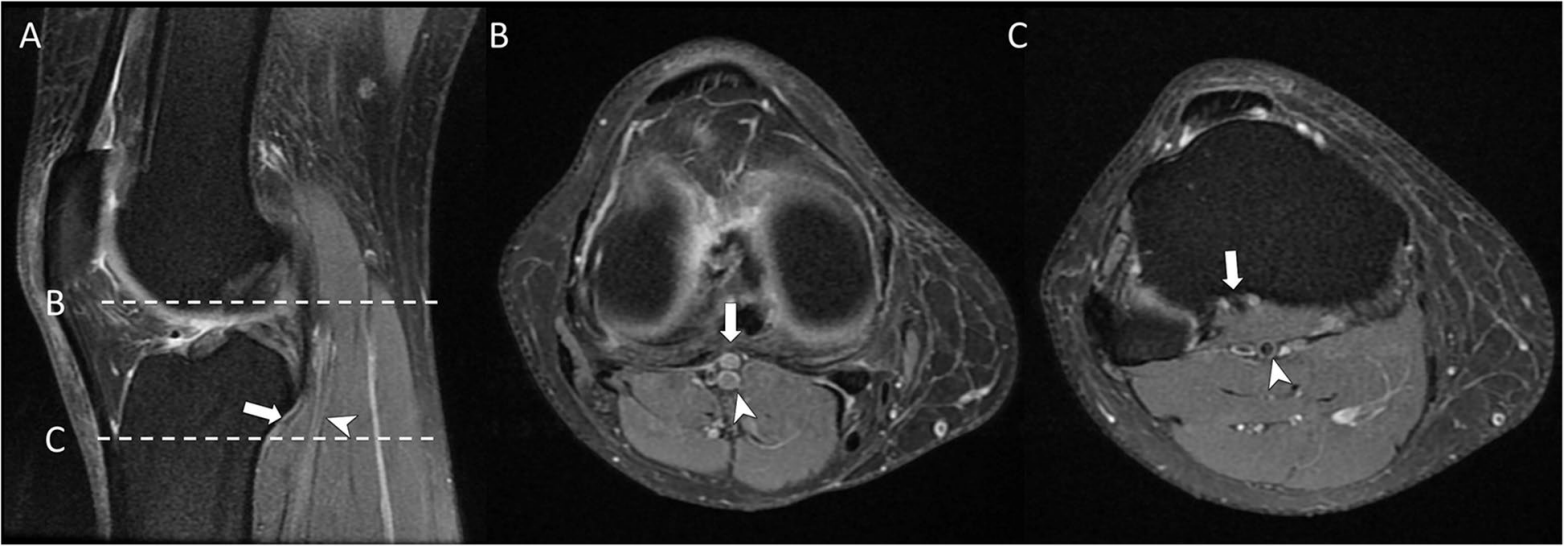
A patient (age range 25–45) with an aberrant anterior tibial artery. **a** Sagittal PDFS shows the distribution of the popliteal artery (arrowhead) and the aberrant anterior tibial artery (arrow) that are separated by the popliteus muscle. **b** Axial PDFS image shows the origin of the aberrant anterior tibial artery. **c** Distribution of the aberrant anterior tibial artery and the popliteal artery at the upper part of the tibia

We were able to localize the popliteal vasculature in all cases. Proton density fat-saturated images provided the best contrast resolution between vasculature and the surrounding structures on visual inspection. Axial and sagittal PDFS allowed the identification of a vascular structure anterior to the popliteal muscle and posterior to the tibial cortex to confirm the diagnosis of AATA (Figs. 3 and 4).

An aberrant anterior tibial artery was visualized in 8 of the 280 (2.9%) examinations using PDFS and in sagittal and axial orthogonal planes. The AATA was present in 5 cases in the left extremity and in 3 cases in the right extremity. In the 27 patients with MRI of both knees, the anatomical variant was present in 4 patients and was unilateral. In total, the prevalence of the aberrant anterior tibial artery was 3.2% (8/253) in our population. The images were evaluated with almost perfect agreement by the observers (k = 0.94). A second evaluation resulted in complete agreement regarding the final diagnoses.

### Literature review

**Fig. 5:**
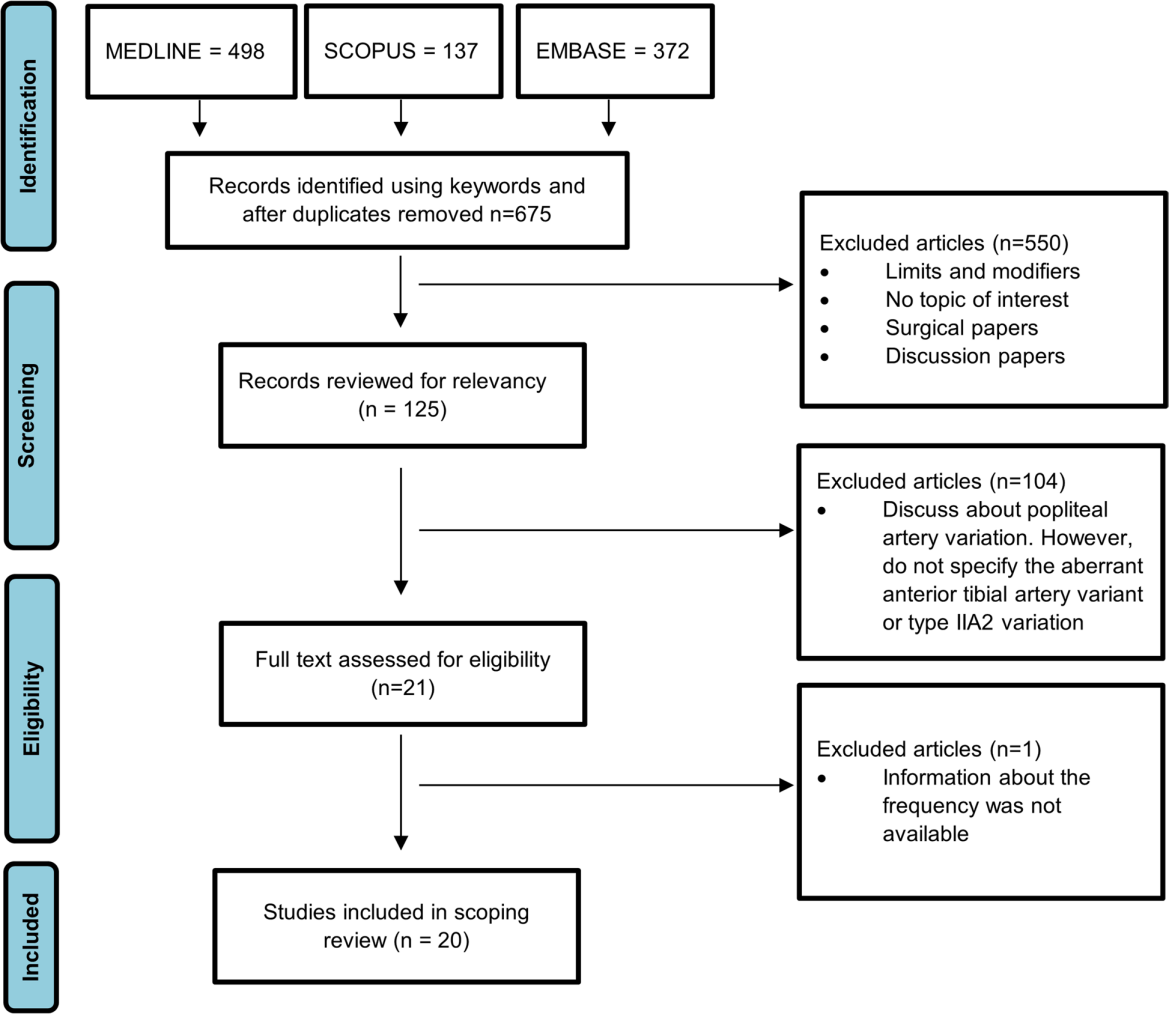
Flow diagram outlining literature search strategy

In our scoping review, we found 20 studies that evaluated and described the presence of the AATA by different methods. Figure 5 describes the identification and screening process used to create the final study list, which identified the studies, designs and study quality described in Table 1. Detailed results of study quality assessement are listed in Supplement 2. Three studies gathered data from limb dissection, 9 from angiograhy, 1 from ultrasound, 6 from CT angiography and 1 from MRI. The frequency of AATA ranged from 0.4 to 6% (median 1%, IQR (0.5–2.3%). Some studies did not provide information about the gender, and 18 of the studies reported on additional anatomical popliteal artery variants besides the AATA.

**Table 1:** Frequency of aberrant anterior tibial artery in the literature

Author	Methods	Extremities Examined	Aberrant Anterior Tibial Artery (%)	Study design	Quality assessment Newcastle-Ottawa Scale
Adachi (1928) [12]	Limb Dissection	770	1	Retrospective observational	5/9
Trotter (1940) [7]	Limb Dissection	1168	2.4	Retrospective observational	5/9
Keen (1961) [13]	Limb Dissection	280	0.4	Retrospective observational	5/9
Kim (1989) [6]	Angiography	600	0.7	Retrospective observational	5/9
Davies (1989) [14]	Angiography	200	1.5	Retrospective observational	5/9
Prayer (1990) [15]	Angiography	414	2.9	Retrospective observational	5/9
Voboril (1990) [16]	Angiography	253	0.4	Retrospective observational	5/9
Day (2006) [17]	Angiography	1037	2.4	Retrospective observational	5/9
Szpinda (2006) [18]	Angiography	152	0.66	Retrospective observational	5/9
Kil (2009) [19]	Angiography	1242	0.4	Retrospective observational	5/9
Mavili (2011) [20]	Angiography	535	1.3	Retrospective observational	5/9
Celtikci (2017) [21]	Angiography	1184	0.6	Retrospective observational	5/9
Tindall (2006) [22]	Doppler Ultrasound	100	6	Prospective consecutive cohort	5/9
Yanik (2015) [8]	CT angiography	126	4.4	Retrospective observational	5/9
Calisir (2015) [23]	CT angiography	742	0.5	Retrospective observational	5/9
Oztekin (2015) [24]	CT angiography	495	0.4	Retrospective observational	5/9
Demirtas (2016) [25]	CT angiography	1261	0.4	Retrospective observational	5/9
Soler (2017) [11]	CT angiography	1633	1.65	Retrospective observational	5/9
Oner (2020) [26]	CT angiography	340	0.6	Retrospective observational	5/9
Klecker (2008) [9]	MRI	1116	2.1	Retrospective observational	5/9
Present cohort	MRI	280	2.9	Retrospective observational	

## Discussion

The main finding of our study is that in a Latin American cohort the AATA could be reliably detected using MRI displaying a relatively high prevalence of 3.2%. This finding is closely similar to the frequency found in a previous angiographic study (2.9%) and a cadaveric study (2.4%) [7]. The only retrospective MRI study that determined the prevalence of AATA reported a frequency of 2.1% in an US-based population [9]. Notably, in the majority of our cases imaging studies were based on unilateral knee evaluation and therefore, bilateral assessment could yield higher prevalence of AATA. A subgroup of 27 patients that had MRI examination in both extremities had a prevalence of 14.8%. This result was higher than previous retrospective angiographics studies that evaluated both extremities and reported lower prevalence < 3.2%. Our results need to be interpreted with caution since the other studies that evaluated both knees enrolled a large number of patients.

**Fig. 6:**
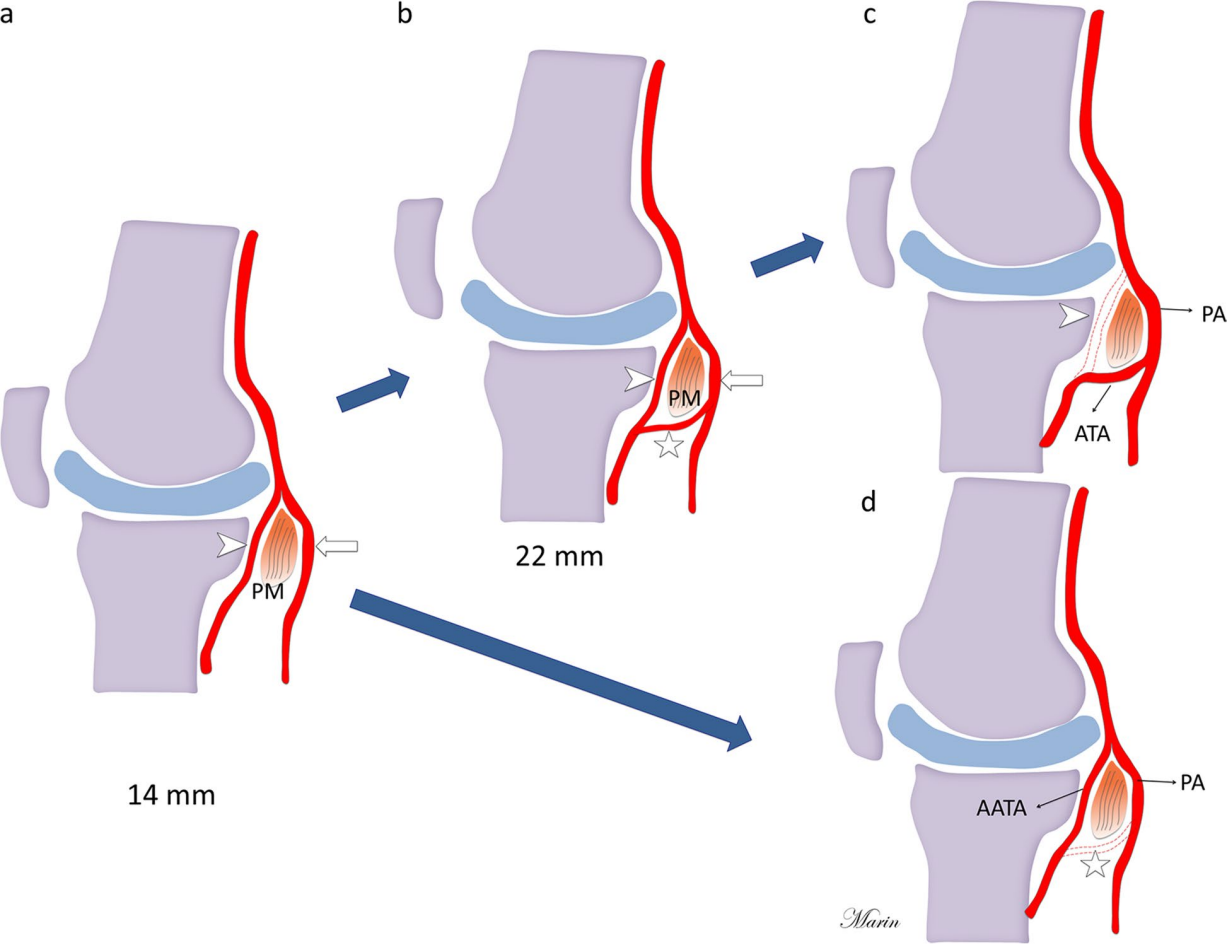
Illustration of the embryological development of the aberrant anterior tibial artery. **a** By 14 mm the deep popliteal artery (arrowhead) courses anterior to the popliteal muscle (PM) and the superficial popliteal artery (arrow) lies posterior to the popliteal muscle. **b** By 22 mm a small connection between both arteries is formed and is called ramus communicans medius (star). **c** This vessel finally becomes the first part of the definitive anterior tibial artery (ATA) in normal cases. **d** Failure in the development of the ramus communicans medius will cause the persistence of the aberrant anterior tibial artery. Figure created by J. Marin-Concha

Anatomical variants of the popliteal artery terminal branching are common. The final distribution of the popliteal artery is defined during the vascular embryologic period. The presence of the AATA is due to the persistence of the poplitea profunda artery and the failure in the development of the ramus communicans medius (Fig. 6) [21, 23, 24, 27, 28]. Historically, variation in the branching of the popliteal artery occurs in about 10% of patients [1, 29]. Lippert and Pabst [30] were among the first to classify the branching of the popliteal arteries according to the order of the splitting of the anterior tibial artery, posterior tibial artery and peroneal artery. Subsequently, Kim et al. [6] evaluated 605 femoral arteriograms and proposed a unified classification system of the popliteal arterial variants based on angiographic appearance (Type I: Normal Branching, Type II: high division of the poplietal artery and Type III: Hypoplastic or aplastic branching with altered distal supply). A subcategory of the high division of the popliteal artery, Type II, is when the tibial artery arises at or above the knee joint. If this vessel courses posterior to the popliteal muscle, it is called Type IIA1, but if the anterior tibial artery courses between the anterior surface of the popliteal muscle and posterior tibial cortex, it is called Type IIA2 or AATA.

Cadaveric studies were among the first to report this anatomical variation [17, 30, 31]. The advantage of this method is that it allows a complete analysis of all the anatomical popliteal variants. A retrospective cadaveric study that evaluated 770 extremities in a Japanese population found that the AATA was present in 1% of the cases [31]. Another retrospective cadaveric study in a U.S population that evaluated 1168 extremities reported a frequency of 2.4% of the Type IIA2 variant [7]. The first imaging modality that was used to evaluate the popliteal branching was angiography. The presence of the AATA is suspected when the vessel takes a medial course initially before its common lateral course [6]. Different angiographic studies in European and South Korean populations reported frequencies that range from 0.4 to 2.9% [12–16, 18, 29, 32]. Doppler ultrasound has been shown to be a precise method to evaluate the AATA despite its limitation assessing the entire knee vascular anatomy. A prospective study that evaluated 100 extremities using Doppler ultrasound showed that the prevalence of AATA was 6% [20]. The advantage of CT angiographic over other methods is the complete and rapid identification of vascular and soft tissue structures. Most of the CT studies were retrospectives observational studies carried out in a European population showed frequency ranging from 0.4 to 4.4% [8, 19, 22, 25, 26, 33]. In contrast with the previous imaging methods MRI appear as an excellent modality because it allows the complete analysis of the articular, vascular and soft tissue knee structures and is included in the mayority of presurgical knee evaluation. To our knowledge there is only one previous retrospective observational cohort that evaluated 1116 extremities using MRI showing a prevalence of 2.1%. The study was done in an American population and reported that the AATA was most easily identified on axial and sagittal planes using PDWI [9]. We did not include this sequence in our knee MRI protocol institution, but we believe that sagitall and axial images are fundamental for the diagnosis.

Presurgical identification of anatomical variations such as the AATA can decrease the risk of vascular trauma [2, 34]. This is especially true in surgical orthopedic procedures such as knee joint surgery, total knee replacement, femoral distal or proximal tibial/fibular fracture surgery, high tibial osteotomy, lateral meniscal repair, posterior ligament cruciate reconstruction, and vascular interventions in popliteal artery aneurysm [5, 35]. Vascular injury can occur due to direct laceration, retraction and following the placement of screws during surgery [3, 4, 36–41]. The region between the tibial cortex and major vessels (popliteal vein, popliteal artery and anterior tibial artery), also called “the danger zone”, carries more than 20% of injury frequency during screw fixation in knee arthroplasty [41].

During orthopedic knee surgical procedures, the popliteal muscle has an important role because it protects the majority of vascular structures. However, in cases of AATA, the artery loses the protection of the popliteal muscle and is vulnerable due to the close relationship with the tibial cortex [35]. Interestingly, patients with a normal vascular distribution of the anterior tibial artery had a lower incidence of peripheral vascular disease than patients with an aberrant variation in a retrospective angiography study [16].

As a limitation our study did not evaluate both knees in the majority of cases due the fact that MRI is usually ordered to answer a specific problem. In our scoping review we found that 75% of the studies (angiographic and computed tomography studies) take into consideration both extremities showing no difference with the unilateral studies [8, 11, 23–26].

## Conclusions

Precise anatomical characterization of the AATA using MRI is valuable in planning invasive interventional procedures of the lower leg since this anatomical variant was present in 8 out of 253 patients in our Latin American cohort. The prevalence of the AATA found in the included studies of the scoping review was 1% IQR (0.5–2.3%).

## Supplementary Information

**Additional file 1.** Search Strategy Example: Ovid (Medline).

**Additional file 2.** Quality assessment (Newcastle Ottawa Scale).

## Data Availability

The datasets used and/or analyzed during the current study are available from the corresponding author on reasonable request.

## Abbreviations

AATA: Aberrant Anterior tIbial Artery
MRI: Magnetic resonance imaging
PDFS: Proton density fat-saturated
TR: Repetition time
TE: Time to Echo
NOS: Newcastle-Ottawa Scale
CT: Computer tomography
