# Supplementary material for: Prevalence and characteristics of the aberrant anterior tibial artery: a single-center magnetic resonance imaging study and scoping review
Source: BMC Musculoskelet Disord. 2021 Nov 2;22:922. doi: 10.1186/s12891-021-04801-9 (PMC8564972; doi:10.1186/s12891-021-04801-9)
Supplement: Supplementary file 1 — Additional file 1. Search Strategy Example: Ovid (Medline). [file 12891_2021_4801_MOESM1_ESM.docx]

Supplement 1. Search Strategy Example: Ovid (Medline)

**Search Strategy Article Retrieved**

1. knee.mp.  300818
2. knee*.mp. 342500
3. vasculature.mp.  175537
4. 1 or 2 342500
5. 3 and 4 6041
6. magnetic resonance imaging.mp.  457219
7. 5 and 6 1569
8. popliteal artery.mp. 9258
9. 7 and 8 167
10. ultrasound.mp.  584740
11. 5 and 10 1987
12. 8 and 11 314
13. computer tomography.mp.  12868
14. 5 and 13 69
15. 8 and 14 7
16. angiography.mp.  275948
17. 5 and 16 1500
18. 8 and 17 399
19. 9 or 12 or 15 or 18 498
