# Supplementary material for: Prevalence and characteristics of the aberrant anterior tibial artery: a single-center magnetic resonance imaging study and scoping review
Source: BMC Musculoskelet Disord. 2021 Nov 2;22:922. doi: 10.1186/s12891-021-04801-9 (PMC8564972; doi:10.1186/s12891-021-04801-9)
Supplement: Supplementary file 2 — Additional file 2. Quality assessment (Newcastle Ottawa Scale). [file 12891_2021_4801_MOESM2_ESM.docx]

**Supplement 2**

Quality assessment (Newcastle Ottawa Scale)

| **Reference** | **Selection** | **Comparability** | **Exposure / Outcome** | **Total** | **Overall**  **assessment** |  |
| --- | --- | --- | --- | --- | --- | --- |
| Adachi (1928) | *** | - | ** | 5/9 | Fair | |
| Trotter (1940) | *** | - | ** | 5/9 | Fair | |
| Keen (1961) | *** | - | ** | 5/9 | Fair | |
| Kim (1989)  Davies (1989)  Prayer (1990)  Voboril (1990)  Day (2006)  Szpinda (2006)  Kil (2009)  Mavili (2011)  Celtikci (2017)  Tindall (2006)  Yanik (2015)  Calisir (2015)  Oztekin (2015)  Demirtas(2016)  Soler (2017)  Oner (2020)  Klecker (2008) | ***  ***  ***  ***  ***  ***  ***  ***  ***  ***  ***  ***  ***  ***  ***  ***  *** | *  -  *  *  *  *  *  *  *  -  *  *  *  *  *  *  * | **  **  **  **  **  **  **  **  ***  **  **  **  **  **  ***  ***  *** | 6/9  5/9  6/9  6/9  6/9  6/9  6/9  6/9  7/9  5/9  6/9  6/9  6/9  6/9  7/9  7/9  7/9 | Fair  Fair  Fair  Fair  Fair  Fair  Fair  Fair  Good  Fair  Fair  Fair  Fair  Fair  Good  Good  Good | |
